# Cardiovascular Risk in Chronic Kidney Disease: Role of the Sympathetic Nervous System

**DOI:** 10.1155/2012/319432

**Published:** 2012-08-07

**Authors:** Jeanie Park

**Affiliations:** ^1^Renal Division, Department of Medicine, Emory University School of Medicine, 1639 Pierce Drive, WMB 338, Atlanta, GA 30322, USA; ^2^Research Service, Department of Veterans Affairs Medical Center, Decatur, GA 30033, USA

## Abstract

Patients with chronic kidney disease are at significantly increased risk for cardiovascular disease and sudden cardiac death. One mechanism underlying increased cardiovascular risk in patients with renal failure includes overactivation of the sympathetic nervous system (SNS). Multiple human and animal studies have shown that central sympathetic outflow is chronically elevated in patients with both end-stage renal disease (ESRD) and chronic kidney disease (CKD). SNS overactivation, in turn, increases the risk of cardiovascular disease and sudden death by increasing arterial blood pressure, arrythmogenicity, left ventricular hypertrophy, and coronary vasoconstriction and contributes to the progression renal disease. This paper will examine the evidence for SNS overactivation in renal failure from both human and experimental studies and discuss mechanisms of SNS overactivity in CKD and therapeutic implications.

## 1. Introduction

Patients with chronic renal failure are at profoundly higher risk for cardiovascular (CV) morbidity and mortality. Multiple epidemiologic studies have demonstrated that both reduced renal function and proteinuria are significantly and independently associated with an increased risk of CV death [1–5]. In multivariate adjusted analyses, patients with end-stage renal disease (ESRD) had a 10 to 20-fold higher risk of CV mortality [3–5]. Such increases in CV risk are not limited to patients with the most advanced renal disease on dialysis. Those with mild-to-moderate chronic kidney disease (CKD) are also at significantly higher risk of CV disease [1, 2], and patients with both overt proteinuria as well as microalbuminuria without a reduction in estimated glomerular filtration rate (eGFR) are also at significantly higher risk [2]. Thus, reduced renal function and proteinuria are powerful and independent risk factors for CV disease and mortality.

The nature of CV disease in patients with renal failure differs from that of the general population. Whereas the most common manifestations of CV disease in the general population include coronary atherosclerotic disease, patients with renal failure are far more likely to suffer from chronic heart failure and sudden cardiac death [3, 6]. Sudden cardiac death is the leading cause of death in ESRD patients, and accounts for approximately 25% of all deaths in this population [7]. In addition, the clinical presentation of CV disease is also different amongst those with renal disease when compared to the general population. For example, CKD patients with acute myocardial infarction are more likely to present with atypical symptoms and shortness of breath than with more typical symptoms [8]. Thus, the pathophysiology of increased CV disease risk in CKD likely differs from that of the general population and may include factors that are specific to the diseased or ischemic kidneys.

**Figure 1:**
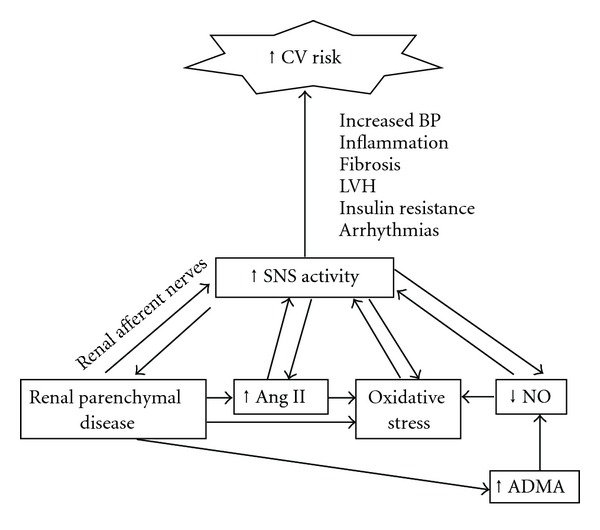
Pathogenesis of sympathetic overactivity in chronic kidney Disease factors contributing to increased sympathetic nervous system (SNS) activity in chronic renal failure, leading to increased cardiovascular (CV) risk. ADMA refers to asymmetric dimethylarginine. NO refers to nitric oxide. Ang II refers to angiotensin II. LVH refers to left ventricular hypertrophy.

The mechanisms underlying CV risk in CKD are multifactorial and begin early in the course of renal disease. Not only are traditional CV risk factors such as hypertension and diabetes highly prevalent in the CKD population, but nontraditional risk factors specific to CKD and ESRD patients are also highly prevalent and contribute to risk [9]. These include oxidative stress, inflammation, decreased nitric oxide (NO) bioavailability, anemia, extracellular volume overload, fluid and electrolyte shifts, malnutrition, abnormal calcium and phosphorus metabolism, infection, uremic toxins, as well as sympathetic nervous system (SNS) overactivity. Multiple human and animal studies have clearly demonstrated that CKD is a state of SNS overactivity [10–13]. SNS overactivity contributes to increased risk of CV disease and sudden death by increasing blood pressure, arrythmogenicity, left ventricular hypertrophy, coronary vasoconstriction, and end-organ damage [14, 15]. Moreover, sympathetic overactivity is directly associated with an increased risk of CV mortality in patients with ESRD [16] and correlates with left ventricular hypertrophy (LVH) in this population [17]. The remainder of this paper will focus on mechanisms by which SNS overactivity increases CV risk, the evidence for SNS overactivation in renal failure, potential mechanisms of chronic sympathoexcitation in CKD (Figure 1), and therapeutic considerations.

## 2. SNS Overactivity Accelerates CV Risk and**** End-Organ Damage

 Epidemiologic studies have demonstrated that SNS overactivity is independently associated with an increased risk of cardiovascular mortality in patients with renal failure and other disease states [14, 16, 18, 19]. The mechanisms by which chronic SNS overactivation leads to increased CV risk have not been fully elucidated. One potential mechanism is indirect, via increased blood pressure induced by alpha-adrenergic vasoconstriction that leads to an increase in total peripheral resistance. However, there is evidence to suggest that SNS activation itself plays a direct role in increasing CV risk and may accelerate end-organ damage at the level of the heart, vasculature, and kidney [14, 18–20]. SNS overactivity is closely linked to the development and progression of chronic heart failure (CHF) [21, 22]. Patients with CHF have elevated levels of plasma NE and MSNA [22, 23], and in animal models, chronic infusion of norepinephrine caused increases in left ventricular weight [19, 24], that were independent of changes in blood pressure, implicating a causal link between SNS overactivity and LVH. In addition, SNS activity has been shown to have a proinflammatory and profibrotic effect on the heart and vasculature [14, 19, 25, 26]. At the level of the vasculature, SNS activation leads to structural changes including arteriolar remodeling and vascular hypertrophy [14, 18, 27], as well as functional reductions in arterial distensibility [28]. SNS activation has also been shown to increase vascular oxidative stress [29], increase arrhythmogenicity [15], and contributes to hyperinsulinemia and insulin resistance [30], further mechanisms by which SNS overactivity contributes to increased CV risk.

 Chronic SNS activation likely also plays a role in the progression of chronic kidney disease. Sympathetic innervation of the kidney increases renovascular tone, and leads to activation of the renin-angiotensin aldosterone system, and increased sodium reabsorption, that contributes to extracellular fluid volume expansion and increased BP. SNS activation also contributes to progression of glomerulosclerosis and proteinuria [14, 20, 31, 32]. In subtotally nephrectomized rats, the central sympatholytic drug, moxonidine, given at nonhypotensive doses reduced renal sympathetic nerve activity with concomitant reduction in glomerulosclerosis [20]. In humans, nonhypotensive doses of moxonidine improved urinary albumin excretion in diabetics and hypertensive patients [31, 32]. Together, these data provide evidence that SNS activation contributes to increased CV risk and progression of renal disease, via both BP-dependent and independent mechanisms at the level of the vasculature, myocardium, and kidney.

## 3. Evidence for Chronic SNS Overactivation in Renal Failure

Studies in humans and animals have provided convincing evidence that chronic renal failure is characterized by SNS overactivity. Plasma norepinephrine levels have been shown to be significantly elevated in patients with ESRD, roughly two-fold higher than in controls [33, 34], and are also elevated in hypertensive CKD patients [35]. Human studies using direct measurements of sympathetic nerve activity directed to muscle (MSNA) via microneurography have shown that SNS activity is chronically elevated in patients with ESRD [13, 36, 37]. SNS overactivity is a feature of earlier stages of CKD as well, and in a stepwise fashion, MSNA increases with worsening eGFR [10, 11, 35, 38]. In an animal model of chronic renal failure induced via 5/6 nephrectomy, the increase in blood pressure was accompanied by increases in NE turnover in areas of the brain responsible for sympathetic regulation, suggesting that elevated BP in CKD is in part mediated via SNS activation [39, 40]. In addition, renal injury without a reduction in eGFR appears to be sufficient in leading to SNS overactivity. In rats, phenol injection into the lower pole of one kidney induces a minimal renal injury without a reduction in eGFR, but results in an immediate increase in blood pressure, NE turnover in the brain, and renal sympathetic nerve activity [41–43]. Similarly, hypertensive humans with polycystic kidney disease (PCKD) but without reduction in eGFR had significantly increased levels of MSNA [44].

## 4. Mechanisms of SNS Overactivity in CKD

### 4.1. Renal Afferent Nerves

The kidney functions as a sensory organ that is richly innervated with chemoreceptors and baroreceptors [45]. These renal afferent nerves connect with centers in the brain that regulate blood pressure and central sympathetic outflow. Animal studies have highlighted the importance of the renal afferent nerves in the pathogenesis of SNS overactivity and high blood pressure in renal failure. In 5/6th nephrectomized rats, the increase in blood pressure was accompanied by increased NE turnover in the areas of the brain responsible for sympathetic regulation, including the posterior, anterior, and lateral hypothalamic nuclei and the locus ceruleus [39]. In animal models, selective renal afferent nerve denervation can be performed with a dorsal rhizotomy, in which the dorsal roots from T10-L2 are severed, selectively denervating the renal afferent nerves while leaving the efferent nerves intact. Dorsal rhizotomy in 5/6 nephrectomized rats prevented the increase in BP and NE turnover in the posterior and lateral hypothalamic nuclei and the locus ceruleus, suggesting that renal afferent nerve activation by the diseased kidneys modulates central SNS activity in rats with chronic renal failure [39]. Furthermore, in the phenol renal injury model of minimal renal damage without a reduction in renal function, the elevation in blood pressure is concomitant with increased NE turnover in sympathetic brain centers, and increase in renal sympathetic nerve activity [41–43]. Again, renal afferent denervation by dorsal rhizotomy normalized blood pressure, NE turnover in the brain, and renal sympathetic nerve activity, in the phenol renal injury model. Thus, renal injury, independent of renal function, activates central SNS outflow via renal afferent nerve activation.

 Human studies using direct recordings of MSNA via microneurography corroborate the findings from experimental studies. In hemodialysis patients with intact kidneys, MSNA was significantly elevated when compared to controls [13]. However, after bilateral nephrectomies, dialysis patients had a significant reduction in MSNA to the level of controls, suggesting a role for the diseased kidneys in chronic SNS overactivation in ESRD. Similarly, restoration of renal function through kidney transplantation resulted in no change in MSNA in transplant recipients with intact native kidneys, independent of treatment with calcineurin inhibitors [46]. However, after undergoing bilateral nephrectomies, MSNA was reduced significantly in transplant recipients to the same levels as controls, suggesting that the diseased kidneys themselves, independent of renal function, contribute to sympathetic overactivity in ESRD.

The specific factors that bind and activate the renal nerves are unknown and largely unstudied. Purported ligands include urea, and adenosine, a product of renal ischemia. Chemoreceptor activation via ischemic metabolites may cause reflex increases in SNS outflow in order to attempt to redirect blood flow away from other areas of the body, to the ischemic tissues. In a study of uninephrectomized dogs, changes in blood pressure and renal sympathetic nerve activity were measured during intrarenal infusion of adenosine, before and after renal denervation [47]. There was a concomitant increase in blood pressure, efferent renal sympathetic nerve activity, and renal NE spillover during adenosine infusion, that was eliminated after renal afferent denervation, implicating a role for adenosine in renal nerve stimulation.

### 4.2. Decreased Nitric Oxide (NO) Bioavailability and Asymmetric Dimethylarginine (ADMA)

Decreased NO bioavailability likely contributes to chronic SNS overactivation in CKD. NO has a tonic inhibitory effect on central SNS outflow. In rats that were chronically treated with N Nitro L arginine methyl ester (L-NAME), an inhibitor of NO synthase (NOS), blood pressure increased due to decreased NO bioavailability [48]. When microinjections of the inhibitory amino acid glycine were injected into the rostral ventral lateral medulla (RVLM), an area of the brain that contains sympathetic premotor neurons important for the control of sympathetic vasomotor tone, there was a reduction in blood pressure during NOS inhibition to the same degree as controls. These data suggest that the increase in blood pressure during NOS inhibition was not entirely due to impaired endothelium-mediated vasodilation, but also due to increased central sympathetic activity. Human studies have also demonstrated that there is an important SNS component to the blood pressure raising effects of NOS inhibition [49, 50]. Alpha adrenergic blockade with phentolamine at doses that had no effect on baseline blood pressure attenuated the increase in blood pressure during NOS inhibition by 40% [49]. Decreased NO bioavailability in humans induced by pharmacological inhibition of NOS caused large increases in blood pressure that are in part mediated by SNS overactivation [49, 50].

 ESRD and CKD patients have reduced NO bioavailability due to multiple causes that include oxidative stress, inflammation, uremic toxins, and accumulation of endogenous inhibitors of NOS, including asymmetric dimethylarginine (ADMA). ADMA is an endogenous inhibitor of NOS that accumulates in CKD and ESRD patients and is not removed during dialysis [51–56]. The mechanisms underlying the accumulation of ADMA in patients with ESRD and CKD include a reduction in renal elimination (i.e., a uremic toxin), enhancement of ADMA production, and impaired degradation of ADMA due to reduced levels of dimethylarginine dimethylaminohydrolase (DDAH), an enzyme that metabolizes more than 90% of circulating ADMA [52, 54, 55, 57]. Infusion of ADMA increased renal sympathetic nerve activity in conscious rats after removal of the influence of arterial baroreceptors [58]. In humans, ADMA levels have been shown to correlate with both MSNA and plasma NE levels in CKD and ESRD [51, 56]. In ESRD patients, ADMA correlates with left ventricular hypertrophy and left ventricular dysfunction [59], carotid intima-media thickness [60], and is a strong and independent predictor of mortality and cardiovascular outcomes in hemodialysis patients [61]. Together, these data suggest that the accumulation of ADMA plays a crucial role in reduced NO bioavailability in patients with chronic renal failure, leading to increased central SNS outflow and its deleterious effects.

### 4.3. Oxidative Stress

Patients with CRF have elevated baseline oxidative stress which contributes to increased CV risk [62–64]. Oxidative stress is involved in the regulation of SNS activity [65]. The generation of reactive oxygen species (ROS) was increased in the RVLM of hypertensive rats, leading to increased central SNS outflow and blood pressure [66]. Oxidative stress in the brain led to sympathoexcitation and increases in arterial BP in rats with salt-sensitive hypertension [67], renovascular hypertension [68], and salt-induced CKD [69]. In the phenol renal injury model, the rise in blood pressure was accompanied by increased NE turnover as well as increased markers of oxidative stress in key brain centers [42]. Blood pressure and sympathetic nerve activity were reduced with the injection of tempol and superoxide dismutase in the lateral ventricle of these rats [42]. In addition to direct central effects, oxidative stress contributes to SNS overactivity by reducing NO bioavailability [70].

### 4.4. Angiotensin II (Ang II)

High circulating Ang II levels also likely contribute to sympathetic activation in CKD. Circulating Ang II can easily reach the area postrema of the medulla which lacks a blood brain barrier and has a multitude of AngII receptors [71]. The area postrema is directly connected to CV control centers in the brain that regulate SNS outflow. In addition to its central effects, Ang II resets the arterial baroreflex at a higher blood pressure level and enhances the action of NE at the sympathetic nerve terminal by increasing the release and decreasing the reuptake of NE. In human studies, infusion of both Ang II and phenylephrine decreased MSNA due to increases in BP leading to baroreflex-mediated inhibition of sympathetic outflow [72]. However, for the same degree of rise in BP, there was less of a reduction in MSNA with Ang II than with phenylephrine. Furthermore, when differences in BP response were eliminated with concomitant nitroprusside infusions, thereby unloading sympathetic baroreceptors, a significantly greater increase in MSNA during Ang II infusion was revealed. Furthermore, clinical studies have shown that blockers of the renin angiotensin system, including angiotensin converting enzyme inhibitors (ACE-i), angiotensin receptor blockers (ARBs), and direct renin inhibitors, reduce MSNA in patients with CKD [35, 38, 73]. These findings suggest that Ang II has a stimulatory effect on central SNS activity.

## 5. Therapeutic Considerations

Clearly, patients with ESRD and CKD have SNS overactivity that contributes to increased risk of CV disease and sudden death, as well as progression of renal dysfunction. However, whether treatment with sympatholytic agents reduces CV risk or modifies the rate of end-organ damage in CKD is yet unknown. Inhibitors of the renin-angiotensin system, including ACE-inhibitors, ARBs, and direct renin inhibitors, have been shown to significantly reduce MSNA in patients with CKD [35, 38, 73]. As these agents are often indicated in CKD patients for treatment of proteinuria and comorbid conditions such as heart failure, in addition to the added beneficial effect on SNS activity, these medications should be highly considered in the treatment of hypertension in this patient population. Whether peripheral sympatholytics, such as beta blockers, are beneficial in CKD is controversial [74, 75]; however, there may be a role for treatment with vasodilating beta blockers such as carvedilol, labetalol, and nebivolol in this patient population. Statins have been shown to reduce MSNA, without a concomitant reduction in blood pressure, in patients with CKD [76]. It is unclear if such reductions are clinically meaningful and will result in long-term improvements in blood pressure or cardiovascular risk in patients with chronic renal failure.

In ESRD patients, short daily dialysis has been shown to reduce ambulatory blood pressure, nocturnal blood pressure dip, and MSNA when compared to conventional hemodialysis [77]. The mechanisms by which short daily dialysis might improve SNS overactivity are unclear, but may include improved clearance of uremic toxins, decreased blood pressure variability, and improved extracellular volume control. Extracellular volume overload may contribute to SNS overactivity in CKD and ESRD by causing nocturnal rostral fluid shifts and peripharyngeal edema [78], resulting in obstructive sleep apnea (OSA), a highly prevalent condition in the ESRD population [79]. Multiple studies have shown that muscle sympathetic nerve activity (MSNA) is chronically elevated in patients with OSA [80–82], and more recently, a significant correlation was found between OSA and resistant hypertension among ESRD patients [83]. Adequate dialysis, volume control, and treatment of obstructive sleep apnea should be standard practice and may help ameliorate SNS overactivation in ESRD.

Finally, newer nonpharmacologic approaches to the treatment of hypertension, particularly resistant or difficult to control hypertension, target reduction of central SNS outflow and may become applicable in the treatment of hypertension in patients with renal disease. These include renal denervation and baroreceptor stimulation. Renal denervation is performed via transcatheter ablation of both afferent and efferent renal nerves and has been shown to substantially reduce blood pressure and MSNA in patients with resistant hypertension [84–86]. The electric baroreflex stimulator implanted at the level of the carotid sinus significantly reduced blood pressure, MSNA, and plasma NE levels in patients with treatment-resistant hypertension. However, both the renal denervation studies and carotid baroreflex stimulator studies excluded patients with reduced renal function. Whether these therapies will have a role in the treatment of hypertension in patients with CKD remain to be tested.

## 6. Future Directions

Patients with CKD and ESRD have chronic sympathetic overactivity that contributes to the substantially increased risk of CV disease and mortality [10, 11, 13, 16, 17]. Despite recent advances, many gaps still exist in our understanding of sympathetic regulation in patients with renal failure. The role of the diseased kidneys themselves in SNS overactivation has been established in experimental and human studies [13, 39, 40, 43]. However, the molecular mechanisms by which reflex activation of central SNS outflow occurs in renal parenchymal disease remains unknown. Identifying the ligands that activate the renal afferent nerves, and the specific renal sensory nerve endings (chemoreceptors, baroreceptors) that are sensitized and evoke an increase in SNS activity may help lead to new therapeutic targets to block this pathway and ameliorate SNS hyperactivity. At the clinical level, interventions such as exercise therapy and dietary modification have been shown to reduce SNS overactivity in other patient populations [23, 87, 88] and may have similar benefits in CKD. Drugs that have anti-inflammatory properties such as statins and bardoxolone, an antioxidant inflammatory mediator that has recently been shown to have beneficial effects in diabetic patients with CKD [89], may also have beneficial effects on SNS activation in patients with renal disease. The effects of new and existing therapies on SNS activity, and the long-term effects on CV risk and progression of renal disease, should be investigated in future studies.
